# G_i_ Proteins Regulate Adenylyl Cyclase Activity Independent of Receptor Activation

**DOI:** 10.1371/journal.pone.0106608

**Published:** 2014-09-09

**Authors:** Caroline Bull Melsom, Øivind Ørstavik, Jan-Bjørn Osnes, Tor Skomedal, Finn Olav Levy, Kurt Allen Krobert

**Affiliations:** 1 Department of Pharmacology, Faculty of Medicine, University of Oslo and Oslo University Hospital, Oslo, Norway; 2 K.G. Jebsen Cardiac Research Centre and Center for Heart Failure Research, Faculty of Medicine, University of Oslo, Oslo, Norway; University of Torino, Italy

## Abstract

**Background and purpose:**

Despite the view that only β_2_- as opposed to β_1_-adrenoceptors (βARs) couple to G_i_, some data indicate that the β_1_AR-evoked inotropic response is also influenced by the inhibition of G_i_. Therefore, we wanted to determine if G_i_ exerts tonic receptor-independent inhibition upon basal adenylyl cyclase (AC) activity in cardiomyocytes.

**Experimental approach:**

We used the G_s_-selective (R,R)- and the G_s_- and G_i_-activating (R,S)-fenoterol to selectively activate β_2_ARs (β_1_AR blockade present) in combination with G_i_ inactivation with pertussis toxin (PTX). We also determined the effect of PTX upon basal and forskolin-mediated responses. Contractility was measured *ex vivo* in left ventricular strips and cAMP accumulation was measured in isolated ventricular cardiomyocytes from adult Wistar rats.

**Key results:**

PTX amplified both the (R,R)- and (R,S)-fenoterol-evoked maximal inotropic response and concentration-dependent increases in cAMP accumulation. The EC_50_ values of fenoterol matched published binding affinities. The PTX enhancement of the G_s_-selective (R,R)-fenoterol-mediated responses suggests that G_i_ regulates AC activity independent of receptor coupling to G_i_ protein. Consistent with this hypothesis, forskolin-evoked cAMP accumulation was increased and inotropic responses to forskolin were potentiated by PTX treatment. In non-PTX-treated tissue, phosphodiesterase (PDE) 3 and 4 inhibition or removal of either constitutive muscarinic receptor activation of G_i_ with atropine or removal of constitutive adenosine receptor activation with CGS 15943 had no effect upon contractility. However, in PTX-treated tissue, PDE3 and 4 inhibition alone increased basal levels of cAMP and accordingly evoked a large inotropic response.

**Conclusions and implications:**

Together, these data indicate that G_i_ exerts intrinsic receptor-independent inhibitory activity upon AC. We propose that PTX treatment shifts the balance of intrinsic G_i_ and G_s_ activity upon AC towards G_s_, enhancing the effect of all cAMP-mediated inotropic agents.

## Introduction

According to conventional understanding, G proteins transduce signals from activated G protein-coupled receptors (GPCRs) via second messengers to regulate numerous downstream signalling targets in the cell [1], [2]. G proteins are found on the cytoplasmic side of the plasma membrane, composed of a guanine nucleotide binding α subunit (Gα) and a βγ dimer (Gβγ). Upon GPCR activation, GDP is exchanged for GTP on Gα, and both the GTP-liganded Gα and the Gβγ dimer regulate downstream targets. Five subfamilies of Gα have been classified, whereof Gα_i/o_ is the only one able to inhibit adenylyl cyclase (AC) and the production of cAMP [3].

The predominant G_i_-coupled receptor in the heart ventricle is the M_2_ muscarinic acetylcholine receptor [4]. Constitutive activity of this receptor exerts a mild, continuous inhibition of AC activity in normal rat ventricular cardiomyocyte membranes [5]–[7]. It is well established that the muscarinic system also antagonizes the inotropic responses mediated by β-adrenoceptors (βARs) [4], known as accentuated antagonism [8], [9].

The major stimulatory input in the myocardium comes from the β_1_- and β_2_ARs, whereby activation of these receptors leads to positive inotropic responses. In 1995, Xiao et al. made the intriguing observation that pertussis toxin (PTX), known to cause ADP-ribosylation of the Gα_i_ subunit, uncoupling the GPCRs from G_i_, enhanced β_2_AR- but not β_1_AR-mediated positive inotropic responses in isolated rat cardiomyocytes [10]. Together with subsequent studies [11]–[14], it has been widely accepted that in addition to G_s_, the β_2_ARs also couple to G_i_ in native systems while the β_1_ARs do not.

However, we recently reported that the isoproterenol-mediated inotropic response in left ventricular muscle strips was potentiated by PTX [15]. When examined separately, both the β_1_- and β_2_AR-mediated inotropic response (βAR-IR) and cAMP accumulation in isolated ventricular cardiomyocytes were increased by PTX pre-treatment [16]. Additional data from rat [17], guinea-pig [18] and normal [19] as well as transgenic mice overexpressing the β_2_AR [20] indicate that β_1_AR-evoked contractility in the heart may also be regulated by G_i_ activity. Due to conflicting data on the role of G_i_ in β_1_AR signalling, we wanted to further investigate this issue.

It has previously been reported that AC activity evoked by forskolin and agonists at G_s_-coupled receptors can be increased by prior PTX treatment in various cell types [21]–[24], as well as in cardiomyocytes [25] and sarcolemmal membranes from failing human myocardium [26]. However, these studies did not take into account the presence of constitutively active GPCRs which are known to regulate basal AC activity [7], [27]. It has been suggested by El-Armouche et al. [28], and shown by Rau et al. [17] through overexpression of Gα_i2_ as well as by Hussain et al. [15] by the use of phosphodiesterase (PDE) 3 and 4 inhibitors that G_i_ may have receptor-independent effects, whereby it directly inhibits AC activity. We examined the possibility that G_i_ may have receptor-independent effects in the absence of constitutively active receptors by using the unique properties exhibited by stereoisomers of the β_2_AR agonist fenoterol. (R,R)-fenoterol was first characterized by Woo et al. (2009) to selectively activate only the G_s_ pathway of the β_2_AR, whereas the other stereoisomers, including (R,S) used in this study, activate both the G_s_ and G_i_ pathways [29]. On this basis, (R,R)-fenoterol has been used as a tool to differentiate G_s_- and G_i_-coupling of the β_2_AR [30]. We further studied receptor-independent effects of G_i_ by using the direct AC activator forskolin, as well as the effects of PDE3 and 4 inhibition in the absence and presence of known antagonists or inverse agonists.

Our data indicate that in addition to the traditional role of G_i_ in receptor signalling, this G protein exerts a constant intrinsic inhibition upon AC independent of receptor activation.

## Methods

### Animal care

Experiments and animal care were conducted in accordance with the European Convention for the protection of vertebrate animals used for experimental and other scientific purposes (Council of Europe no. 123, Strasbourg 1985) and approved by the Norwegian Animal Research Authority. The animals were housed with a 12/12 h cycle at 21°C, food and water available *ad libitum*.

### Measurement of contractility in left ventricular strips

Left ventricular strips (diameter ∼1.0 mm) were prepared from male Wistar rats (Taconic, Skensved, Denmark) weighing 300–350 g, mounted in 31°C organ baths containing physiological salt solution with 1.8 mM Ca^2+^, equilibrated and field-stimulated at 1 Hz [31], [32]. Contraction-relaxation cycles were recorded and analysed as previously described [33], [34]. Maximal development of force (dF/dt)_max_ was measured and inotropic responses were expressed as increase in (dF/dt)_max_. Concentration-response curves were constructed by estimating centiles (EC_10_ to EC_100_) and calculating the corresponding means, and the horizontal positioning is expressed as –logEC_50_ values [33].

**Figure 1:**
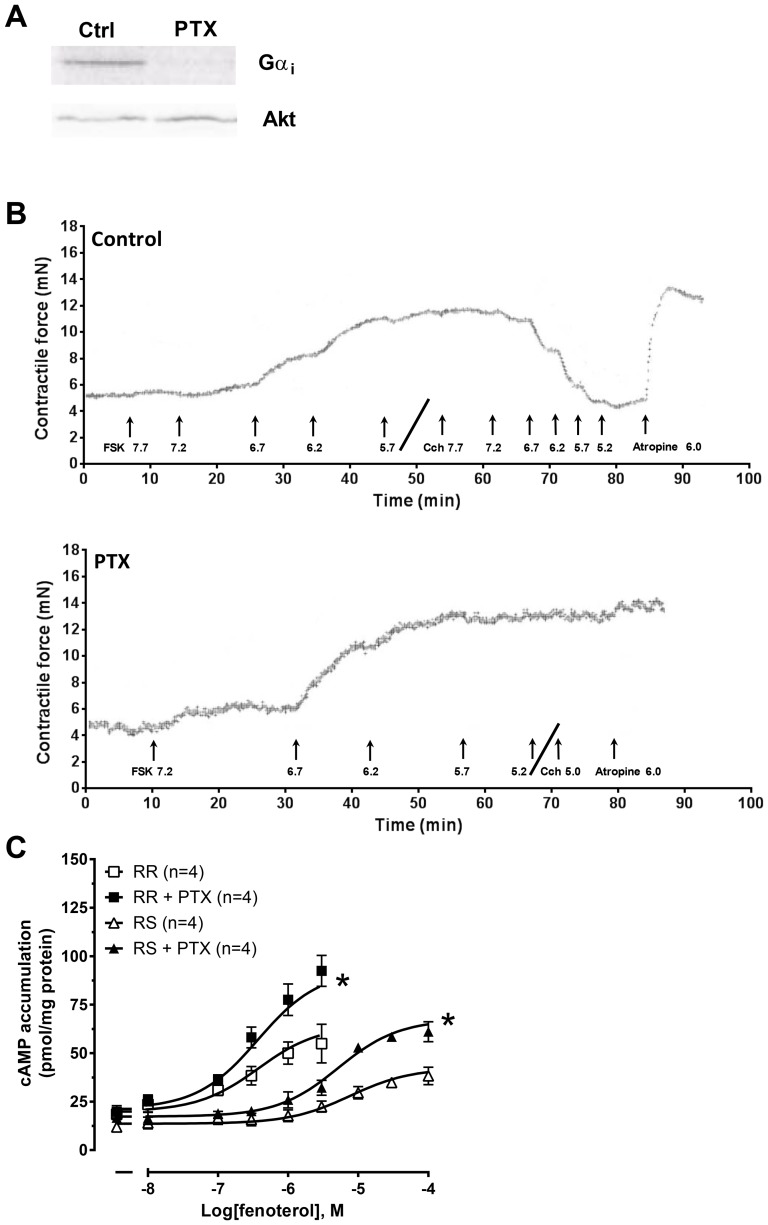
Effect of PTX upon (R,R)- and (R,S)-fenoterol-induced cAMP accumulation. (A) Representative autoradiogram showing ADP-ribosylated Gα_i_ protein levels in rat ventricle pre-treated with saline (control) or PTX. (B) Representative traces showing inotropic responses (mN) evoked by forskolin (FSK) and the subsequent effect of carbachol (Cch) and reversal of carbachol effects by atropine in left ventricular strips from rats pre-treated with saline (Control; top) or with PTX (bottom). Drug concentrations are given in -Log(M). (C) Concentration-response curves to (R,R)- and (R,S)-fenoterol-mediated cAMP accumulation in isolated ventricular cardiomyocytes in the presence of IBMX and the β_1_AR antagonist CGP20712 (300 nM) in control or after PTX pre-treatment. Data are mean ± SEM. Basal cAMP accumulation was (in pmol cAMP/mg protein): (R,R-series) control: 18.5±2.8; PTX: 19.7±3.2; (R,S-series) control 11.9±2.5; PTX 16.7±2.0. RR: (R,R)-fenoterol; RS: (R,S)-fenoterol; *P<0.05, paired t-test.

In a subset of rats, PTX was administered at a dose of 60 µg/kg i.p. as a single injection three days prior to isolation of the left ventricular muscle strips. Control rats were given a saline injection of equal volume. Data from animals treated with PTX were only included if carbachol inhibition of the βAR-IR was completely abolished (Fig. 1B). To confirm the effectiveness of PTX treatment *in vivo* (subset of 6 PTX-treated rats and 7 saline-treated rats) and in cardiomyocytes (see below), we measured the level of PTX-catalysed incorporation of [^32^P]ADP-ribose from [^32^P]NAD into available G_i_ as previously described [15].

### Measurement of cAMP accumulation in left ventricular cardiomyocytes

Adult left ventricular cardiomyocytes were isolated from excised male Wistar rat hearts by retrograde aortic perfusion with a nominally Ca^2+^-free JOKLIK-MEM solution and enzymatic digestion using collagenase (90 U/mL) as previously described [35]. Left ventricular cardiomyocytes were incubated for 20 h in the absence or presence of 1 µg/ml PTX (1.2 ml reaction volume). Experiments were conducted in the presence of the β_1_AR blocker CGP20712 (300 nM), the non-selective PDE inhibitor IBMX (0.5 mM) or PDE3 and 4 inhibitors cilostamide (1 µM) and rolipram (10 µM), as indicated. cAMP accumulation was measured by radioimmunoassay as previously described [36]. Protein was measured with the Coomassie Plus Protein Assay (Pierce, Rockford, IL) according to the manufacturer's protocol and cAMP accumulation was normalized to the amount of protein in each sample.

### Measurement of (R,R)- and (R,S)-fenoterol-mediated inotropic responses and ability to activate adenylyl cyclase

To evaluate the β_2_AR-mediated inotropic response, G_s_-selective (R,R)-fenoterol and G_s_- and G_i_-activating (R,S)-fenoterol were assessed by conducting a concentration-response experiment in the presence of a selective β_1_AR antagonist (300 nM CGP20712). Likewise, (R,R)- and (R,S)-mediated activation of AC was assessed by incubating cardiomyocytes for 10 min with increasing concentrations of either stereoisomer (0–100 µM) in the presence of 300 nM CGP20712. All cAMP accumulation experiments in this subset were conducted in the presence of the non-selective PDE inhibitor IBMX (0.5 mM).

### Measurement of spontaneous intrinsic G_i_ inhibition upon adenylyl cyclase activity

Receptor-independent G_i_ activity was assessed through forskolin concentration-response curves (50 nM-10 µM) in left ventricular strips and isolated cardiomyocytes in the presence of the non-selective βAR antagonist timolol (1 µM) and α_1_-adrenoceptor antagonist prazosin (0.1 µM, ventricular strips only) with and without prior pre-treatment with PTX (as described above). To determine if spontaneous intrinsic activity of G_i_ regulated basal AC activity, the inotropic response in ventricular strips or cAMP accumulation in cardiomyocytes pre-treated with or without PTX was measured in the presence of both the PDE3 inhibitor cilostamide (1 µM) and PDE4 inhibitor rolipram (10 µM) in the presence of the non-selective βAR antagonist timolol (1 µM) and α_1_-adrenoceptor antagonist prazosin (0.1 µM, ventricular strips only).

### Statistics

Data are expressed as mean ± SEM from n animals. p<0.05 was considered statistically significant (One-way ANOVA and student's t-test). When appropriate, Bonferroni corrections were made to control for multiple comparisons.

### Drugs and solutions

Prazosin hydrochloride, (-)isoprenaline hydrochloride, timolol maleate, atropine sulphate, lidocaine (2-diethylamino-N-[2,6-dimethylphenyl]-acetamide) hydrochloride, L-ascorbic acid and 3-isobutyl-1-methylxanthine (IBMX) were purchased from Sigma-Aldrich (St. Louis, MO, USA). CGP20712 dihydrochloride, CGS 15943, cilostamide and rolipram were purchased from Tocris Bioscience (Bristol, UK). Forskolin was from LC Laboratories (Woburn, MA, USA). (R,R)- and (R,S)-fenoterol were a kind gift from J. Kozocas (SRI International, Menlo Park, CA, USA). Isoflurane (1-chloro-2,2,2-trifluoroethyl difluoromethyl ether; Forene) was from Abbot Scandinavia (Solna, Sweden). Pertussis toxin was from Merck chemicals (Nottingham, UK). [^3^H]cAMP was from PerkinElmer (Boston, MA, USA).

## Results

### G_i_ was essentially inactivated by pertussis toxin

The effectiveness of *in vivo* PTX treatment to inhibit G_i_ was assessed by measuring PTX-catalysed incorporation of [^32^P]ADP-ribose from [^32^P]NAD into available G_i_ in ventricular tissue. An ∼80-90% reduction was seen in the ability of subsequent PTX to ADP-ribosylate G_i_ *in vitro* in animals pre-treated with PTX compared to animals given saline injection (PTX-treated n = 6, saline-treated control n = 7, see Fig. 1A). It was not possible to distinguish the different Gα_i_ isoforms in the autoradiogram due to the similarity in size (39-41 kDa). Importantly, in the PTX-treated group, data were included only if carbachol-induced inhibition of the βAR-IR was abolished (Fig. 1B). In the (R,R)- and (R,S)-fenoterol study, 83% of the PTX-treated rats were included in the study. In the forskolin and PDE inhibitor study 88% of the rats met the criteria and were included.

### (R,R)- and (R,S)-fenoterol-stimulated cAMP accumulation was amplified after inactivation of G_i_

(R,R)- and (R,S)-fenoterol-induced AC activation was assessed by measuring cAMP accumulation in isolated left ventricular cardiomyocytes in the presence of the non-selective PDE inhibitor IBMX. Surprisingly, PTX inactivation of G_i_ amplified the concentration-response curve to G_s_-selective (R,R)-fenoterol (Fig. 1C). (R,R)-fenoterol selectively activated the β_2_AR at the concentration range 10 nM-3 µM, after which it overcame β_1_AR blockade by CGP20712 (not shown). Maximal cAMP accumulation evoked by 3 µM (R,R)-fenoterol was enhanced by 60% from 55 pmol/mg protein in control to 92 pmol/mg protein in the presence of PTX. The EC_50_ was not significantly different after PTX treatment (140±48 nM in control and 245±22 nM after PTX treatment) (Fig. 1C). These EC_50_ values were similar to the previously published binding affinity of (R,R)-fenoterol at the β_2_AR (350 ± 30 nM [37]).

As expected, (R,S)-fenoterol concentration-response curves were similarly modified by PTX treatment. Maximal cAMP accumulation evoked by the highest selective concentration of (R,S)-fenoterol for the β_2_AR (100 µM) was enhanced by 62% from 38 pmol/mg protein in control to 61 pmol/mg protein in the presence of PTX. The EC_50_ value was not significantly shifted from 3.5±0.5 µM in control to 5.0±0.9 µM after PTX treatment (Fig. 1C). Again, these EC_50_ values were similar to the published binding affinity of (R,S)-fenoterol of 3.7 ± 0.3 µM [37]. Together, these data demonstrate that PTX amplifies the amount of cAMP produced by both stereoisomers, despite dissimilar G protein-coupling profiles. This suggests that G_i_ exerts inhibitory activity upon AC downstream of receptor coupling to G protein.

### Maximal inotropic responses to both (R,R)- and (R,S)-fenoterol were amplified by G_i_ inactivation or PDE inhibition

**Figure 2:**
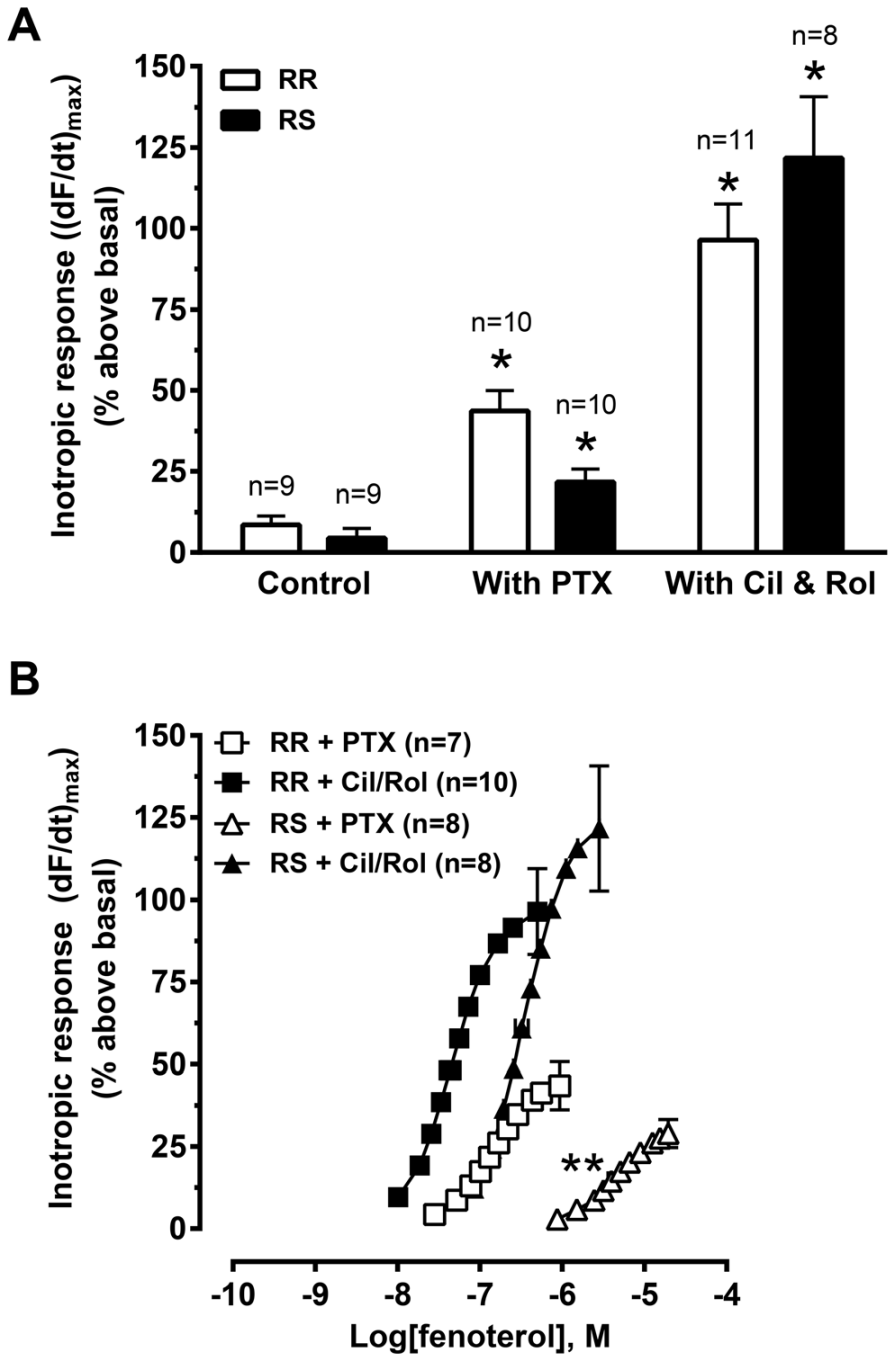
Effect of PTX and PDE3 and 4 inhibition upon the inotropic response to (R,R)- and (R,S)-fenoterol stimulation. Maximal inotropic response (A) and concentration-response curves (B) to (R,R)- or (R,S)-fenoterol in left ventricular strips from saline-treated rats (control) or PTX-treated rats or strips from saline-treated rats given PDE3 (cilostamide, Cil, 1 µM) and PDE4 (rolipram, Rol, 10 µM) inhibitors. All experiments were conducted in the presence of the β_1_AR blocker CGP20712 (300 nM). Inotropic responses are expressed as (dF/dt)_max_ as percent above basal. Basal force was (in mN/mm^2^) (R,R) control: 4.3±0.4; (R,S) control: 4.2±0.6; (R,R) PTX: 3.5±0.5; (R,S) PTX: 3.5±0.4; (R,R) with Cil/Rol: 3.6±0.3; (R,S) with Cil/Rol: 3.9±0.4. Data are mean ± SEM. RR: (R,R)-fenoterol; RS: (R,S)-fenoterol; *P<0.05 vs. control, One-way ANOVA with Bonferroni adjustment for multiple comparisons. **P<0.05 vs. (R,S) with Cil/Rol, One-way ANOVA with Bonferroni adjustment for multiple comparisons.

In untreated (control) left ventricular muscle strips, the highest concentration given of (R,R)- and (R,S)-fenoterol elicited very small inotropic responses of 8.6±2.7% and 4.4±2.9% above basal, respectively (β_1_AR antagonist CGP20712 present; Fig. 2A). These values were slightly lower than inotropic responses obtained by β_2_AR stimulation by adrenaline in the presence of CGP20712 (∼11% above basal, data not shown).

The inotropic responses evoked by maximal concentrations of both (R,R)- and (R,S)-fenoterol were significantly amplified by either PTX pre-treatment (44±6% or 22±4% above basal, respectively) or PDE3 and 4 inhibition (96±11% or 122±19% above basal, respectively) (Fig. 2A). These data are consistent with the cAMP accumulation data and reinforce the hypothesis that G_i_ may have downstream effects independent of receptor activation in a physiological model using intact, isometrically contracting ventricular muscle. Further, the data reflect that increased cAMP accumulation translates to an increased inotropic response.

In the absence of PTX treatment or PDE inhibition it was not possible to determine the EC_50_ values for either fenoterol isoform, due to the very small inotropic response. However, in the presence of PTX, the EC_50_ of (R,R)-fenoterol was 139±18 nM and of (R,S)-fenoterol 4.1±0.4 µM (Fig. 2B), very similar to that obtained from cAMP accumulation (Fig. 1C) and corresponding to the affinity values reported in the literature [37]. PDE3 and 4 inhibition shifted the concentration-response curves of both stereoisomers to lower concentrations (EC_50_: 58±16 nM for (R,R)- and 0.44±0.01 µM for (R,S)-fenoterol), indicating that both G_i_ and PDE3 and 4 regulate translation of the cAMP signal to a functional inotropic response.

### Forskolin responses are enhanced by inactivation of G_i_

**Figure 3:**
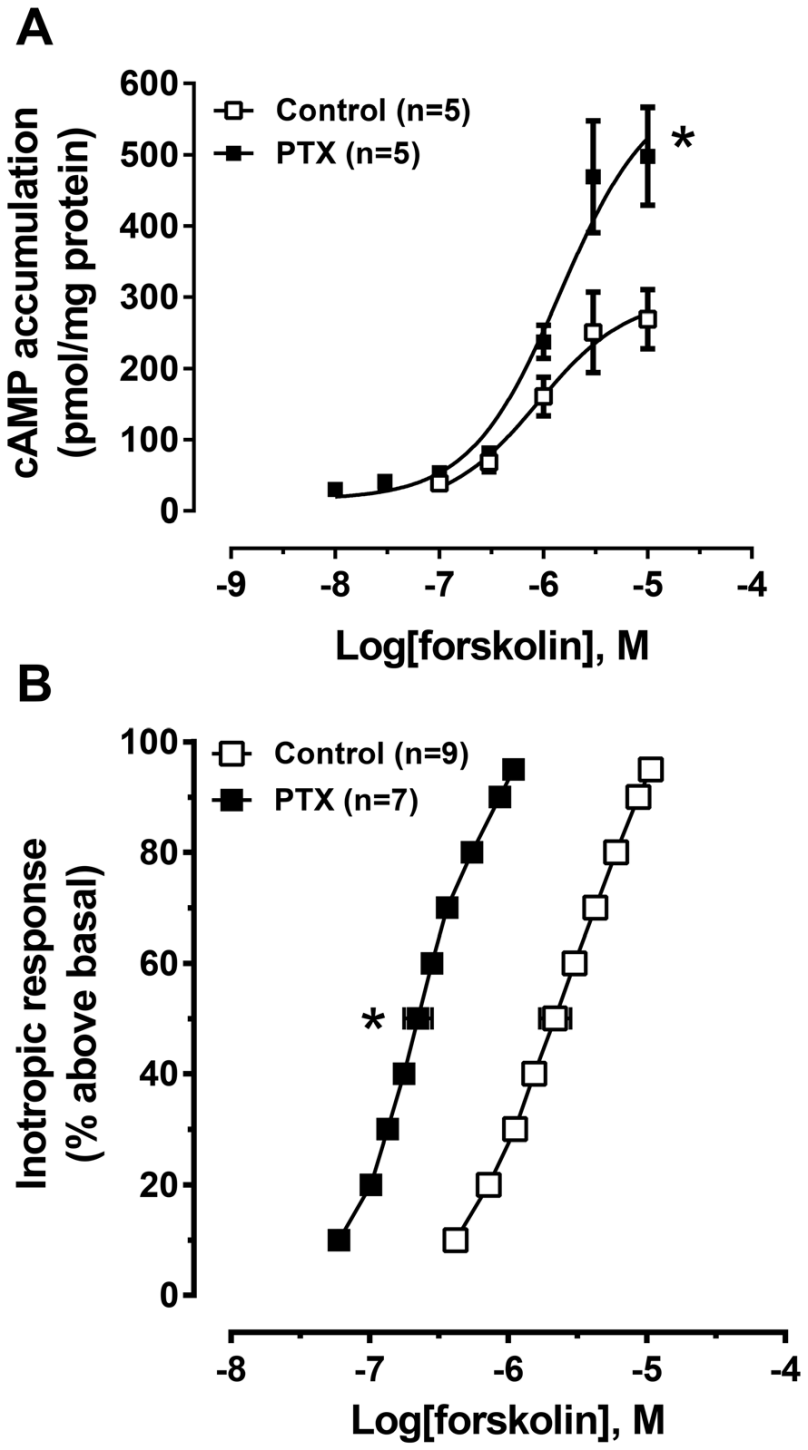
Effect of PTX upon the forskolin-evoked cAMP accumulation and inotropic response. Concentration-response curves of forskolin-evoked cAMP accumulation in ventricular cardiomyocytes (A) and the inotropic response in ventricular strips (B) in the presence of the βAR blocker timolol (1 µM) in control or after PTX pre-treatment. Accumulation of cAMP was measured in the presence of IBMX, with basal cAMP accumulation (in pmol cAMP/mg protein): control: 38.8±5.3; PTX: 30.4±4.0. Basal force was (in mN/mm^2^): control: 4.05±0.56; PTX: 3.91±0.66. Data are mean ± SEM. *P<0.05, paired t-test (cAMP accumulation) or unpaired t-test (inotropic response).

Forskolin, a direct activator of AC, was used to study receptor-independent effects of G_i_ on AC activity. To eliminate the influence of constitutive βAR activation of G_s_ (known to enhance forskolin responses), all experiments were conducted in the presence of the βAR inverse agonist timolol. cAMP accumulation in response to the maximum concentration of forskolin tested (10 µM; this concentration was sufficient to reach an asymptotic response) was enhanced by 85% by PTX treatment from 269±41 pmol/mg protein in control to 498±69 pmol/mg protein in the presence of PTX (Fig. 3A).

In control left ventricular strips, forskolin (10 µM) evoked an inotropic response 120±15% above basal with an EC_50_ of 2.2 µM. After pre-treatment with PTX, the response to forskolin was significantly shifted to 10-fold lower concentrations, yielding an EC_50_ of 0.22 µM, with no change in the maximum inotropic response (103±20% above basal) at maximal tested concentrations (asymptote reached in range from 1–10 µM, Fig. 3B). Together, these data demonstrate that forskolin-stimulated AC activity and functional response in the absence of G protein-coupled receptor activation is modified by PTX treatment.

### Simultaneous inactivation of PDE3 and PDE4 produced a robust cAMP-dependent inotropic response in myocardium only after prior inactivation of G_i_

**Figure 4:**
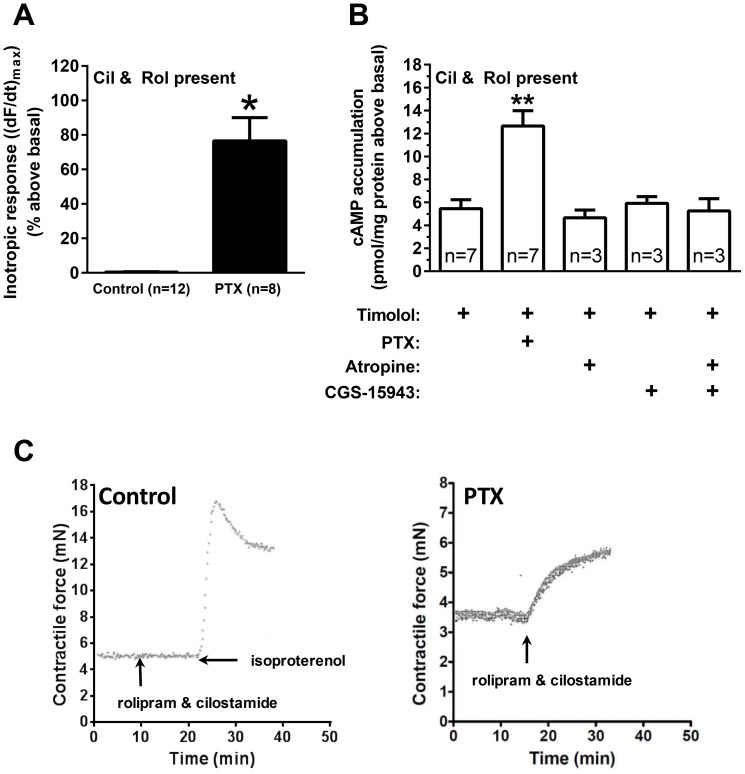
Effect of PTX upon PDE3 and PDE4 inhibitor-induced cAMP accumulation and inotropic response. (A) Effect of simultaneous inhibition of PDE3 (cilostamide, 1 µM) and PDE4 (rolipram, 10 µM) to evoke an inotropic response in left ventricular strips in control (open bars) and after PTX pre-treatment (solid bars) in the presence of timolol (1 µM). Basal force was (in mN/mm^2^): control: 3.3±0.4; PTX: 3.1±0.3. (B) Effect of PTX, βAR inverse agonist timolol (1 µM), non-selective muscarinic inverse agonist atropine (1 µM) or non-selective adenosine receptor inverse agonist CGS 15943 (1 µM) upon PDE3 (cilostamide, 1 µM) and PDE4 (rolipram, 10 µM)-evoked cAMP accumulation. Basal cAMP accumulation (in pmol/mg protein): timolol control: 4.8±0.6 (n = 7). (C) Representative traces showing inotropic responses (mN) evoked by inactivation of PDE3 and 4 simultaneously (1 µM cilostamide and 10 µM rolipram) in left ventricular strips of saline-treated control (left) and after PTX pre-treatment (right) in the presence of 1 µM timolol and 0.1 µM prazosin. Isoproterenol (100 µM displacing timolol) was given to demonstrate that an inotropic effect could be elicited through βARs. Data are mean ± SEM. * P<0.05 PTX vs. control, unpaired t-test; ** P<0.05 vs. timolol, One-way ANOVA with Bonferroni adjustment for multiple comparisons.

The following experiments were conducted in the presence of βAR blockade (timolol, 1 µM), to eliminate a possible effect of residual endogenous noradrenaline or constitutive activation of G_s_ through β_1_- or β_2_ARs. As previously reported [15], and replicated in this study, concomitant PDE3 and PDE4 inhibition (1 µM cilostamide and 10 µM rolipram) was sufficient to elicit a large inotropic response in ventricular strips (77±14% above basal) after PTX treatment (Fig. 4A,C right trace). However, simultaneous inhibition of PDE3 and 4 did not cause an inotropic effect in control hearts (0.42±0.19% above basal) (Fig. 4A,C left trace), indicating the absence of constitutively active G_s_-coupled receptors. To test whether the effect of PTX occurred as a result of removing constitutive receptor activation of G_i_, we evaluated the effect of two established G_i_-coupled receptor systems in the heart. Neither the muscarinic inverse agonist atropine (1 µM) nor the non-selective adenosine inverse agonist CGS 15943 (1 µM), shown to be an inverse agonist at the G_i_-coupled A_1_ adenosine receptor [38], alone or in combination evoked a change in contractile force above basal (Fig. S1).

To investigate the relationship between cAMP and the inotropic response evoked by PDE3 and 4 inhibition after PTX treatment, cAMP accumulation was measured in isolated ventricular cardiomyocytes with or without PTX pre-treatment (Fig. 4B). Simultaneous inhibition of PDE3 and 4 increased cAMP levels in control cardiomyocytes (5.5±0.8 pmol/mg protein above basal). The level of cAMP accumulation evoked by simultaneous inhibition of PDE3 and 4 was significantly increased (∼2-fold) in cardiomyocytes pre-treated with PTX (to 12.7±1.3 pmol/mg protein above basal, Fig. 4B). The effect of PTX was not due to removal of constitutively active G_i_-coupled receptors, since neither atropine (1 µM) nor CGS 15943 (1 µM) alone or in combination in the presence of simultaneous inhibition of PDE3 and 4 mimicked the effect of PTX treatment (Fig. 4B).

## Discussion and Conclusions

Data from this study indicate that G_i_ tonically inhibits basal cAMP production, limiting functional responses. This inhibition appears to be independent of constitutive receptor activation of G_s_ and G_i_ upon AC. In support, (1) PTX treatment increased responses evoked by both the G_s_-selective (R,R)- and the dually coupled (R,S)-fenoterol isoforms (Fig. 1C & 2A); this should not occur if the PTX effect was dependent upon receptor activity, since β_2_ARs stimulated by (R,R)-fenoterol have been shown to only activate G_s_ [29], [39]. (2) PTX treatment amplified forskolin-evoked cAMP accumulation and increased the potency of forskolin to evoke an inotropic response (Fig. 3A,B). Responses to forskolin, being a direct activator of AC [40], should not have been enhanced by inactivation of G_i_; and (3) PTX treatment revealed intrinsic AC activity upon basal responses only after inhibition of PDE3 and 4, whereas inverse agonists at muscarinic receptors and adenosine receptors were without effect after PDE3 and 4 inhibition (Fig. 4A,B). This indicates that PDE3 and 4 inhibition, even in the presence of the βAR inverse agonist timolol, increased cAMP levels in PTX-treated tissue sufficiently to produce an inotropic response (Fig 4A,B,C). If constitutively active G_s_-coupled receptors were mediating this effect, an inotropic response should have occurred after PDE3 and 4 inhibition alone in the absence of PTX treatment. Further, PDE3 and, at least in the rat ventricle, PDE4 activity (Fig. 4A,B; [41]) normally degrades low basal levels of cAMP, maintaining basal contractile force. These data highlight the necessity to remove the tonic inhibition of G_i_ upon AC prior to PDE inhibition to allow for the enhanced cAMP signal to be transduced into a functional response. We propose that PTX removes an intrinsic inhibition of G_i_ upon AC that is independent of G_i_-coupled receptors. That neither the inverse agonist atropine (muscarinic M_2_) nor the non-selective adenosine receptor antagonist CGS 15943, alone or in combination, elicits effects similar to PTX (Fig. 4B, Fig. S1) indicates that PTX does not simply remove the effect of constitutively active G_i_-coupled receptors.

### G_i_ has a propensity to be in a spontaneously active conformation

There is a good basis indicating that G_i_ has receptor-independent activity. Lutz et al. [26] reported that in sarcolemmal membranes from failing human myocardium, G_i_ rapidly released GDP in a time- and temperature-dependent manner. When GDPβS was used in place of GDP to hold G_i_ in the inactive state, AC activity increased, suggesting relief of tonic inhibition. This effect was replicated by Mn^2+^ or high Mg^2+^, which prevent AC inhibition by G_i_. The authors suggest that AC was inhibited by an empty but apparently active G_i_ (even in the absence of activating GTP), further highlighting the inhibitory potential of presumably inactivated G_i_. [26].

Further, Piacentini et al. [42] reported that the synthetic GTP analogue Gpp(NH)p had no effect on basal AC activity. With the G_s_∶G_i_ ratio ranging from ∼1∶10 to 1∶40 [43], [44], G_i_ would be the predominant G protein activated under these conditions. Since AC may already be maximally inhibited by spontaneously active G_i_, it is expected that no additional effect of Gpp(NH)p is seen. However, when the stable GDP analogue GDPβS was used, basal AC activity increased, presumably through relief of tonic G_i_ inhibition. Under this condition, subsequent addition of Gpp(NH)p caused inhibition of AC, presumably through competition with GDPβS [42]. Thus, AC inhibition could be relieved by introduction of synthetic GDP-analogues and re-introduced by synthetic GTP-analogues, providing further support for receptor-independent G_i_ intrinsic activity.

Regulators of G protein signalling (RGS) are GTPase-accelerating proteins that promote rapid GTP hydrolysis and consequently inhibit signal transduction by inactivating G proteins and returning them to the GDP-bound heterotrimeric state. The effect of mutated, RGS-insensitive Gα_i2_ has been studied in cardiomyocytes derived from embryonic stem cells [45] and mice homozygous for the mutated, RGS-insensitive Gα_i2_ [46], providing a model for studying active G_i_. Interestingly, basal inotropic and lusitropic responses were reduced by ∼10% in these mice compared to wildtype [46]. Also, G_s_-dependent stimulation of beating rate by the β_2_-AR agonist procaterol was nearly abolished in foetal RGS-insensitive Gα_i2_-cardiomyoctes via a PTX-sensitive mechanism, further indicating that G_i_ exerts tonic inhibition on AC [45].

Chen-Goodspeed et al. [47] reported that human AC5 has high basal activity when expressed in Sf9 cell membranes, which was concentration-dependently inhibited by GTPγS-activated Gα_i_. AC6 basal activity was low, but could be inhibited by Gα_i_ if previously activated with GTPγS-Gα_s_ [47]. Thus, the two most common AC isoforms in the heart have the tendency to be spontaneously active, providing a substrate for opposition by intrinsic G_i_ activity.

In addition, data suggest that AGS proteins (activators of G protein signalling) may promote G protein activation by either directly acting as a guanine nucleotide exchange factor or by interfering with subunit association/dissociation/trafficking (both dependent and independent of nucleotide exchange) preventing heterotrimeric Gαβγ formation [48]. Together, these effects of AGS could potentially provide another plausible mechanism for mediation of receptor-independent G_i_ activity. Consistent with this hypothesis, Graham et al. [49] have shown that AGS1, which is selective for Gα_i2_ and Gα_i3_, inhibited cAMP accumulation evoked by constitutively active G_s_ or forskolin in 293T cells [49].

### Does PTX treatment shift the balance of intrinsic G_i_ and G_s_ activity upon AC towards G_s_?

Although the current data support the hypothesis that G_i_ exerts intrinsic receptor independent inhibition upon spontaneous AC activity, the mechanism is still unknown. Further, we cannot completely rule out the possibility that PTX treatment removes an unknown constitutively active G_i_-coupled receptor. Despite these limitations, it is reasonable to hypothesize that the ratio of G_s_∶G_i_ has important implications for the regulation of basal AC activity. As reported here, a minimum of 80-90% ADP-ribosylation of G_i_ was necessary to reveal functional effects of PTX treatment in our models. Under this scenario, the normal reported ratio of G_s_∶G_i_ of ∼1∶10 to ∼1∶40 would be altered to ∼1∶1 to ∼1∶4, giving G_s_ more favourable terms to compete for binding to AC. This is consistent with Lutz et al. [26] who suggested that the AC system may be designed to operate from a predominantly off position (high intrinsic G_i_ activity), as both basal AC activity and G_s_-coupled receptor activation would be enhanced by PTX inactivation of G_i_. The mechanism that mediates these effects of PTX treatment remains to be determined.

**Figure 5:**
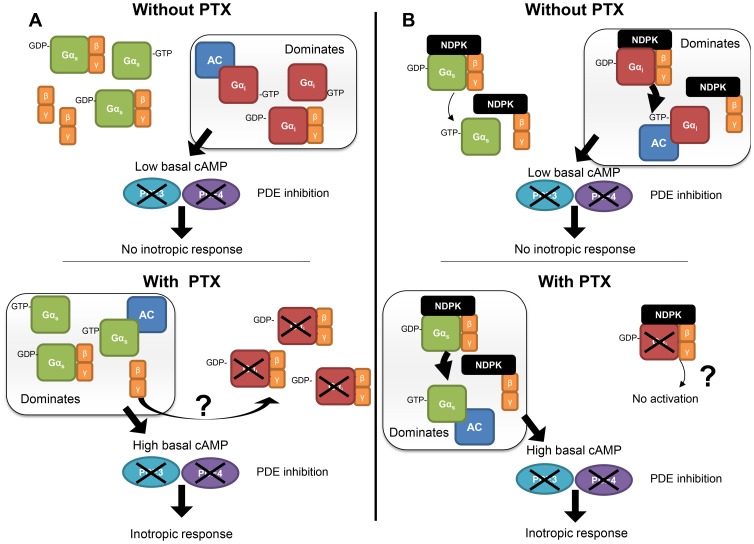
Possible mechanisms mediating the receptor-independent role of G_i_ in regulating basal AC activity. (A) βγ-sink hypothesis: In the absence of PTX (top panel), spontaneously active G_i_ dominates, inhibiting basal AC activity and maintaining low cAMP levels which are incapable of eliciting an inotropic response even after inhibition of PDE3 and 4 (inhibition marked by X). After PTX treatment (bottom panel), G_i_ is not only inactivated (through ADP-ribosylation, marked by X) removing its spontaneous intrinsic inhibition upon AC, but also sequesters a large proportion of the shared Gβγ pool. This indirectly increases the proportion of receptor-independent spontaneously active G_s_, leading to increased basal AC activity and cAMP that is normally readily degraded by PDE3 and 4. However, inhibition of PDE3 and 4 (marked by X) allows for translation of this cAMP increase into an inotropic response (see Fig. 4A). The net result is a shift from predominantly spontaneous G_i_ activity towards G_s_ activity, increasing basal AC activity and promoting activation of AC and positive inotropic effects of all inotropic agents working through increased cAMP signalling. (B) NDPK-hypothesis: In the absence of PTX (top panel), NDPK-activation of G_i_ dominates due to excess of G_i_ protein levels over G_s_, resulting in low cAMP production readily degraded by PDE3 and 4. In the presence of PTX (bottom panel), G_i_ is inactivated by permanent ADP-ribosylation (marked by X). Thus, NDPK B could predominantly activate G_s_, leading to both increased basal contractile force and cAMP accumulation that becomes revealed after PDE3 and 4 inhibition (marked by X).

It has recently been reported that G_s_ and G_i_ compete for an apparently limited pool of βγ [50]. Based on this finding, it is possible that as inactive GDP-bound G_i_ becomes ADP-ribosylated by PTX, G_i_ sequesters βγ from the total shared pool. Under this scenario, over time, there will be less βγ available for G_s_, consequently increasing the probability of G_s_ to be in its active state (GTP-bound) in addition to the corresponding removal of intrinsically active G_i_. A plausible hypothesis would be that G_i_ acts as a βγ sink. In support, overexpression of βARK-ct, a βγ scavenger, resulted in increased AC stimulation [51]. The net effect of PTX treatment would be to shift the balance from largely intrinsic inhibition to a greater intrinsic stimulation upon AC, thereby sensitising AC and enhancing/amplifying those systems known to activate AC (Fig. 5A).

Alternatively, based on the findings of Hippe et al. [52], PTX treatment may shift the ability of endogenous nucleoside diphosphate kinase B (NDPK B) to activate the G_i_ and G_s_ proteins. NDPK B is a G protein histidine kinase that regulates cAMP synthesis and cardiomyocyte contractility independent of GPCRs through the formation of an NDPK B-G_βγ_ complex that activates G protein through a phosphotransfer from NDPK B to His-266 in G_β_ [52]. The phosphate is then transferred onto GDP, and the resultant GTP leads to receptor-independent G protein activation [52]–[54]. In the normal state, due to the excess of G_i_ over G_s_, NDPK B would predominantly activate G_i_ and the stimulatory effects of G_s_ upon basal cAMP generation may be neutralized [52]. After PTX treatment, G_i_ is inactivated by permanent ADP-ribosylation. Thus, NDPK B would predominantly activate (potentiate) G_s_ activity leading to both increased basal contractile force and cAMP accumulation that becomes revealed after PDE3 and 4 inhibition (Fig. 5B). That we also observe enhancement of other cAMP signalling inotropes and forskolin is consistent with this hypothesis. To this end, experiments are currently underway to test both hypotheses, and should provide greater clarity of the mechanism behind intrinsic receptor-independent inhibitory activity of G_i_ upon AC.

## Supporting Information

Figure S1 **Effect of PTX, βAR inverse agonist timolol (1 µM), non-selective muscarinic inverse agonist atropine (1 µM) or non-selective adenosine receptor inverse agonist CGS-15943 (1 µM) upon PDE3 (cilostamide, 1 µM) and PDE4 (rolipram, 10 µM)-evoked inotropic response.** Data are mean ± SEM. * P<0.05 vs. PTX, One-way ANOVA with Bonferroni post test adjustment for multiple comparisons.
(TIF)
