# Supplementary figures and images for: Gi Proteins Regulate Adenylyl Cyclase Activity Independent of Receptor Activation
Source: PLoS One. 2014 Sep 9;9(9):e106608. doi: 10.1371/journal.pone.0106608 (PMC4159282; doi:10.1371/journal.pone.0106608)

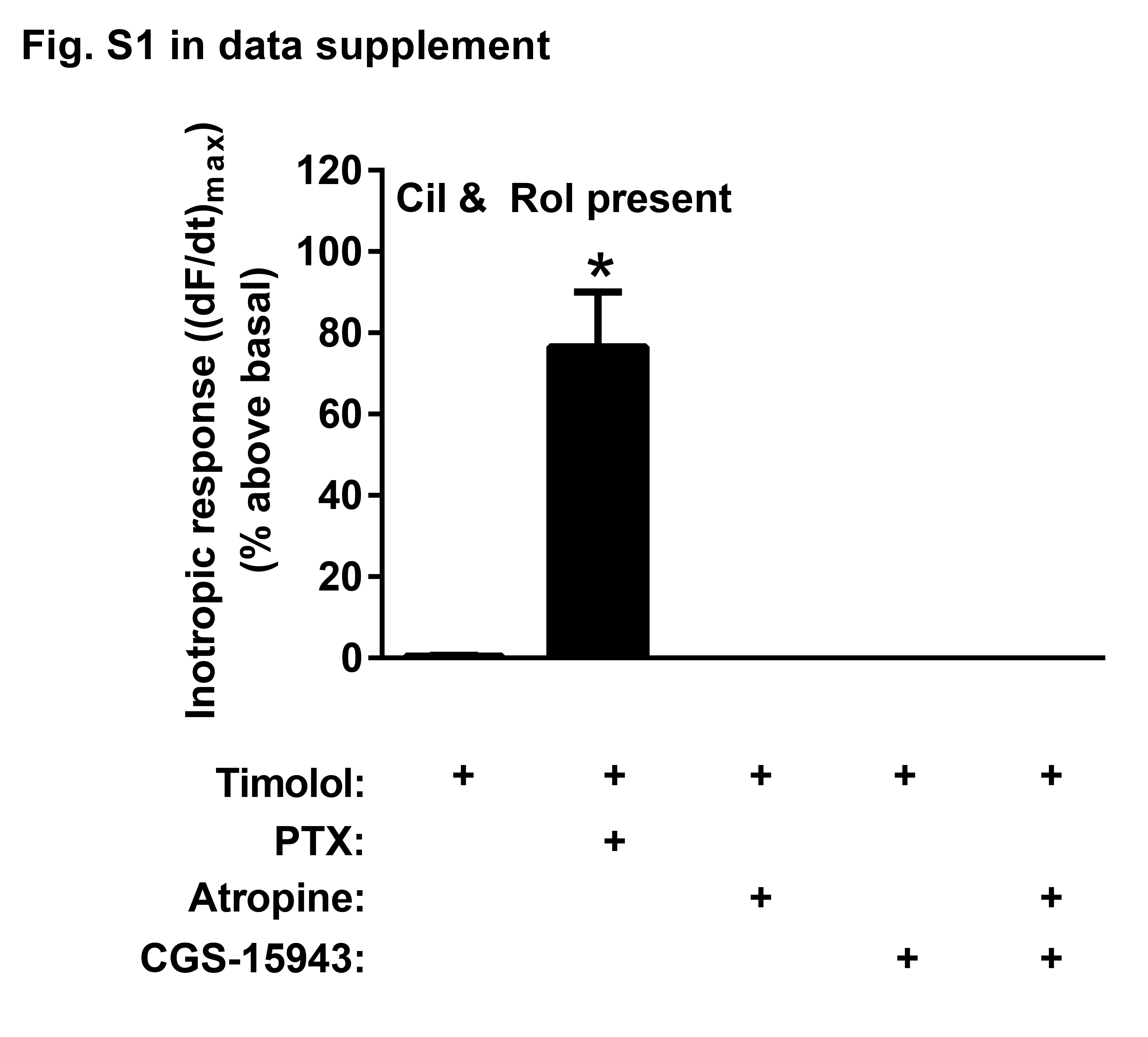

Supplement: Figure S1 — Effect of PTX, βAR inverse agonist timolol (1 µM), non-selective muscarinic inverse agonist atropine (1 µM) or non-selective adenosine receptor inverse agonist CGS-15943 (1 µM) upon PDE3 (cilostamide, 1 µM) and PDE4 (rolipram, 10 µM)-evoked inotropic response. Data are mean ± SEM. * P<0.05 vs. PTX, One-way ANOVA with Bonferroni post test adjustment for multiple comparisons. (TIF) [file pone.0106608.s001.tif]
